# Are Bacterial Volatile Compounds Poisonous Odors to a Fungal Pathogen *Botrytis cinerea*, Alarm Signals to *Arabidopsis* Seedlings for Eliciting Induced Resistance, or Both?

**DOI:** 10.3389/fmicb.2016.00196

**Published:** 2016-02-23

**Authors:** Rouhallah Sharifi, Choong-Min Ryu

**Affiliations:** ^1^Molecular Phytobacteriology Laboratory, Super-Bacteria Research Center, Korea Research Institute of Bioscience and BiotechnologyDaejeon, South Korea; ^2^Department of Plant Protection, College of Agriculture and Natural Resources, Razi UniversityKermanshah, Iran; ^3^Biosystems and Bioengineering Program, University of Science and TechnologyDaejeon, South Korea

**Keywords:** bacterial volatile organic compounds, phytohormones, induced systemic resistance, plant growth-promoting rhizobacteria, biofilm formation, leaf surface attachment

## Abstract

Biological control (biocontrol) agents act on plants via numerous mechanisms, and can be used to protect plants from pathogens. Biocontrol agents can act directly as pathogen antagonists or competitors or indirectly to promote plant induced systemic resistance (ISR). Whether a biocontrol agent acts directly or indirectly depends on the specific strain and the pathosystem type. We reported previously that bacterial volatile organic compounds (VOCs) are determinants for eliciting plant ISR. Emerging data suggest that bacterial VOCs also can directly inhibit fungal and plant growth. The aim of the current study was to differentiate direct and indirect mechanisms of bacterial VOC effects against *Botrytis cinerea* infection of *Arabidopsis*. Volatile emissions from *Bacillus subtilis* GB03 successfully protected *Arabidopsis* seedlings against *B. cinerea*. First, we investigated the direct effects of bacterial VOCs on symptom development and different phenological stages of *B. cinerea* including spore germination, mycelial attachment to the leaf surface, mycelial growth, and sporulation *in vitro* and *in planta*. Volatile emissions inhibited hyphal growth in a dose-dependent manner *in vitro*, and interfered with fungal attachment on the hydrophobic leaf surface. Second, the optimized bacterial concentration that did not directly inhibit fungal growth successfully protected *Arabidopsis* from fungal infection, which indicates that bacterial VOC-elicited plant ISR has a more important role in biocontrol than direct inhibition of fungal growth on *Arabidopsis*. We performed qRT-PCR to investigate the priming of the defense-related genes *PR1*, *PDF1*.*2*, and *ChiB* at 0, 12, 24, and 36 h post-infection and 14 days after the start of plant exposure to bacterial VOCs. The results indicate that bacterial VOCs potentiate expression of *PR1* and *PDF1*.*2* but not *ChiB*, which stimulates SA- and JA-dependent signaling pathways in plant ISR and protects plants against pathogen colonization. This study provides new evidence for bacterial VOC-elicited plant ISR that protects *Arabidopsis* plants from infection by the necrotrophic fungus *B. cinerea*. Our work reveals that bacterial VOCs primarily act via an indirect mechanism to elicit plant ISR, and have a major role in biocontrol against fungal pathogens.

## Introduction

Plants have evolved complex and efficient surveillance systems that respond to abiotic and biotic stresses, including pests and pathogens (Agrios, 2004). Small signaling molecules elicit plant cell defense responses throughout the plant; these include the phytohormones salicylic acid (SA), jasmonic acid (JA), and ethylene (ET) (Pieterse et al., 2009). Each hormone has a particular function in eliciting plant immunity. For example, SA-dependent signaling is triggered by necrotizing avirulent pathogens, whereas JA signaling is triggered by necrotrophic pathogens and insect pests (Mysore and Ryu, 2004). Plants with activated immune systems produce diverse classes of pathogenesis-related (PR) proteins and toxic phenol compounds such as phytoalexins, which can function to induce subsequent defense mechanisms (Ryals et al., 1996; Pieterse et al., 2009; Balmer et al., 2013). Plant immune responses can be induced by pathogens, insects, and beneficial root-associated bacteria designated as plant growth-promoting rhizobacteria/fungi (PGPR/PGPF). PGPR/PGPF systems elicit similar plant immune responses as those elicited by pathogens and insects (Kloepper et al., 2004). Plant resistance to pathogens and insects is largely mediated by the two plant hormones, JA and ET, which elicit induced systemic resistance (ISR) responses (Pieterse et al., 2009; Balmer et al., 2013). Previous studies reported that ISR were effectively elicited by necrotrophic pathogens such as *Pectobacterium carotovorum* (Han et al., 2006; Farag et al., 2013). To identify microbial determinants that elicit ISR, microbial secreted products have been tested on plants under greenhouse and field conditions, including siderophores, phytohormone mimetics, *N*-acyl homoserine lactone, vitamins, and cell wall components such as chitin, glucan, and lipopolysaccharides (Lyon, 2007; Hartmann and Schikora, 2012; Kanchiswamy et al., 2015a,b; Lee et al., 2015).

New investigations clearly demonstrate that PGPR/PGPF emit volatile compounds that trigger robust plant systemic defense responses against pathogenic bacteria (Ryu et al., 2004; Kishimoto et al., 2007; Rudrappa et al., 2010; Song and Ryu, 2013). Ryu et al. (2004) and Rudrappa et al. (2010) shown that bacterial volatile organic compounds (VOCs) activate plant defenses in a strain-specific manner. Ryu et al. (2004) reported that activation of systemic defense in *Arabidopsis* against *Erwinia carotovora* subsp. *carotovora* elicited by *Bacillus subtilis* strain GB03 VOCs depends on ET pathways but was independent of JA and SA pathways. Rudrappa et al. (2010) showed that SA and ET are required for *B. subtilis* FB17 VOC-elicited ISR in *Arabidopsis* against the hemibiotrophic pathogen *Pseudomonas syringae* pv. tomato DC3000, whereas JA was not required. Not only bacteria strains but also a single volatiles can employ different signaling pathways to boost plant defense. Some bacteria emit 2,3-butanediol and its precursor acetoin as VOCs; these volatiles have roles in plant protection against pathogens (Farag et al., 2006). 2,3-butanediol activated *PR-4* expression and SA-dependent signaling in *Agrostis stolonifera* (Cortes-Barco et al., 2010a), application of 100 μM 2,3-butanediol on *Nicotiana benthamiana* seedlings elicited resistance against the hemibiotrophic fungus *Colletotrichum orbiculare* (Cortes-Barco et al., 2010b) by increasing basic PR proteins expression, which are markers of JA-dependent signaling, whereas there was no change in acidic PR proteins expression, which are markers of SA-dependent signaling. 2,3-butanediol activated both SA- and JA-dependent signaling pathways in *Arabidopsis thaliana* in response to abiotic stress (Cho et al., 2008). Currently, there is debate in the literature regarding the function and activity of *Bacillus* VOCs.

Many studies report antagonistic effects of VOCs on plant pathogenic fungi (McCain, 1966; Kai et al., 2007). VOCs can act as antibiotics and directly inhibit mycelial growth and spore germination of pathogenic fungi (Kai et al., 2007; Vespermann et al., 2007). Fiddaman and Rossall (1993) and Chaurasia et al. (2005) show that *B. subtilis* VOCs deform mycelia and inhibit growth of some pathogenic and biocontrol fungi. *B. subtilis* VOCs induce protoplasm retraction in *Botrytis cinerea* hyphae (Chen et al., 2008). However, most of these reports utilize a dual culture method that exposes pathogenic fungi to extremely high VOC levels, which do not occur under natural conditions. Therefore, it is necessary to determine whether *Bacillus* VOCs protect *Arabidopsis* against *B. cinerea* via ISR or by inhibiting fungal growth or infection.

The objective of this work was to investigate direct and indirect mechanisms of *Arabidopsis* protection conferred by *B. subtilis* strain GB03 VOCs against the necrotrophic fungal pathogen *B. cinerea*. First, we evaluated the effects of different VOC concentrations on stages of the *B. cinerea* life cycle and during *Arabidopsis* infection. Secondly, we examined direct and indirect VOC effects on *Arabidopsis* protection against pathogenic fungal infection. Third, we investigated the priming of defense gene expression conferred by bacterial VOCs as a means of eliciting plant ISR. Our results indicate that low concentrations of *B. subtilis* GB03 VOCs induce plant systemic resistance and defense responses, and protect *Arabidopsis* against infection by the necrotrophic pathogen *B. cinerea*. This study broadens the understanding of plant defense mediated by bacterial volatile compounds.

## Materials and Methods

### Evaluating Bacterial Volatile Effects on *Botrytis cinerea* Growth and Plant Protection from Infection

To study the effects of *B. subtilis* GB03 VOCs on *B. cinerea* spore germination, we placed a small Petri dish measuring 40 mm diameter and 10 mm depth inside a larger Petri dish measuring 90 mm diameter and 15 mm depth. Tryptic soy broth (Difco Co, MD, USA) agar (TSA) medium was poured into the larger plate and allowed to solidify. Then, 0–3 sterile filter paper disks (5 mm diameter) were placed around the periphery of the TSA plate. The disks were saturated with a 30 μl suspension of 10^8^ colony forming units (CFU)/ml of *B. subtilis* GB03. The inoculated plates were sealed with lids and incubated at 30°C for 24 h. Then, 4 ml of 1 × 10^5^ CFU/ml of *B. cinerea* spores in half-strength potato dextrose broth (Difco Co, MD, USA) were added to the small 4 cm plate in the center of the inoculated 9 cm plate. The lid of the larger Petri dish was sealed with Parafilm and incubated at 25°C (**Figure 2B**). Spore germination was assessed after 6 h by observing 50 spores per replicate plate at 200× magnification (Nikon Eclipse E600, Osaka, Japan), and percentage germination was recorded. Spores with germinated mycelia longer than half of the spore diameter were considered as germinated.

The effect of bacterial VOCs on *B. cinerea* mycelial growth was investigated using the double-plate assay of Ting et al. (2011). A 9 cm dish containing TSA medium was prepared, 1–3 sterile filter paper disks (5 mm) were placed on the medium, and 30 μl of 10^8^ CFU/ml of *B. subtilis* GB03 suspension was pipetted onto the filter disks. The plates were sealed with lids and incubated at 30°C for 24 h. Then, agar plugs of *B. cinerea* mycelia were taken from the periphery of a plate containing a young growing culture, and the plugs were inserted into the center of a 9 cm Petri dish containing 20 ml of PDA. This plate was inverted and securely fitted over the *B. subtilis* plate. The two plates were sealed together with Parafilm and incubated at 25°C (**Figure 2A**). Radial growth of the fungus was evaluated after 7 days.

The effect of bacterial VOCs on *B. cinerea* sporulation was examined using the same double-plate assay used for the mycelial growth test with slight modification. To evaluate sporulation, a plate was prepared with agar plugs of *B. cinerea* mycelia and incubated at 25°C until vegetative mycelial growth filled the plate. Then, this plate was inverted over the top of a TSA plate containing 1–3 sterile filter paper disks saturated with 30 μl of *B. subtilis* GB03 suspension, the plates were sealed together using Parafilm, and incubated at 25°C for 7 days. Fungal spores were harvested with 10 mM MgCl_2_, and the spore suspensions were passed through gauze cloth to remove mycelial fragments. The numbers of spores produced under each bacterial VOC dosage were counted using a hemocytometer and a compound light microscope.

### Analysis of Mycelial Attachment to Polystyrene Surface

The mycelial surface attachment evaluated in broth medium same as the method used to evaluate fungal spore germination. The layer of mycelium in top of broth medium showing the air-medium interface was photographed after 7 days (**Figure 4**). The surface attachment which reflects hydrophobic surface tendency, was evaluated using the crystal violet (CV) staining method according to Mowat et al. (2007). Briefly, the 4 cm Petri dishes (SPL, Pocheon-si, Gyeonggi-do, South Korea) containing fungi were washed four times with tap water to remove non-attached mycelia and spores. Petri dishes were air dried and attached mycelia were stained with 4 ml of 0.5% CV for 15 min at room temperature. Excess CV stain was removed by washing several times and gently pipetting off the rinsate until excess stain was removed. The remaining CV stain was solubilized by the addition of 4 ml of 95% ethanol. Solubilized CV was gently pipetted off, and the absorbance at OD_600_ was determined using a spectrophotometer (Ultraspec 7000, Biochrom Co, Cambridge, England). Mycelia attachment to leaf surface have been assessed by microscopic inspection of *Arabidopsis* leaves after staining with Trypan blue according to (Koch and Slusarenko, 1990).

### Assessment of ISR in Plants Treated with Bacterial Volatiles

*Arabidopsis* seeds were surface sterilized with 1% (v/v) sodium hypochlorite for 20 min, rinsed three times in sterile distilled water (SDW), and placed on Petri dishes containing half-strength Murashige and Skoog (MS) medium (Duchefa Biochemie, Haarlem, The Netherlands) containing 0.8% (w/v) plant agar and 1.5% (w/v) sucrose (pH 5.8). The plates were placed in a growth cabinet with a 16 h light/8 h dark cycle provided by fluorescent lights (8,000 lux), and temperature was maintained at 22°C with 50–60% relative humidity. Two days after vernalization, seedlings were transferred to specialized plastic Petri dishes that contained a center partition (designated as I-plates). One chamber of the I-plate contained half-strength MS agar medium and the other chamber contained TSA onto which 5 mm diameter sterile filter paper disks had been placed. The seedlings were transferred to the chamber containing MS agar medium. The chamber containing TSA was inoculated with 30 μl of a *B. subtilis* suspension or SDW applied dropwise onto the filter paper disk. Plates were sealed with Parafilm and transferred to the growth chamber using the same conditions. Fourteen days later, 5 μl of *B. cinerea* spore suspensions (10^5^ CFU/ml) were drop-inoculated onto five leaves per *Arabidopsis* seedling. Leaves exhibiting symptoms were determined by visual inspection 3 days after inoculation.

### Differentiating Direct and Indirect Mechanisms of Bacterial VOC-Mediated Plant Protection from *Botrytis cinerea* Infection

These experiments were performed using the I-plate method described by Ryu et al. (2004). Two-day-old *Arabidopsis* seedlings were transplanted into one chamber of an I-plate containing MS agar medium, and 30 μl of 10^8^ CFU/ml *B. subtilis* GB03 suspension or distilled water control was pipetted onto a sterile filter paper disk on TSA medium in the other chamber. After 14 days of seedling exposure to VOCs, the plates were separated into two groups. In one group, the TSA medium and disks containing bacteria were removed from the I-plate (designated as the *B. subtilis*-removed group). In the other group, the TSA medium and disks containing bacteria was allowed to remain in the I-plate (designated as the *B. subtilis*-treated group). Subsequently, all plates were subjected to inoculation with 5 μl of *B. cinerea* spore suspensions (10^5^ CFU/ml) onto five leaves per *Arabidopsis* seedling. Disease severity was evaluated 3 days after pathogen challenge.

Percentage of direct and ISR effect of VOCs on disease suppression calculated based on below formula:

$$Direct\,\;effect=\frac{DS\,\;in\,\;B.\,\;subtilis\,\;removed-DS\,\;in\,\;B.\,\;subtilis\,\;treated}{DS\,\;in\,\;control-DS\,\;in\,\;B.\,\;subtilis\,\;treated}\times 100$$

$$ISR=\frac{DS\,\;in\,\;control-DS\,\;in\,\;B.\,\;subtilis\,\;removed}{DS\,\;in\,\;control-DS\,\;in\,\;B.\,\;subtilis\,\;treated}\times 100$$

Where DS is disease severity percent.

### Evaluation of Plant Defense Priming using Quantitative RT-PCR

Total RNA was isolated from inoculated 14-day-old leaf tissues 0, 12, 24, and 36 h after *B. cinerea* inoculation according to the protocol of Yang et al. (2009). Total RNA was treated with 1 U of RNase-free DNase (Promega, USA) for 10 min at 37°C, and then subjected to a second round of purification using TRI reagent. First-strand cDNA synthesis was performed in 20 μl of AccuPower RT PreMix (Bioneer, Korea) containing 1 μg of DNase-treated total RNA, oligo(dT) primers, and Moloney murine leukemia virus reverse transcriptase (MMLV- RT; Invitrogen, USA). PCR reactions were performed according to the manufacturer’s instructions. The candidate gene was analyzed using the following primers: 5′-GCTTCAGACTACTGTGAACC-3′ (*ChiB_F*) and 5′-TCCACCGTTAATGATGTTCG-3′ (*ChiB_R*); 5′-AATGAGCTCTCATGGCTAAGTTTGCTTCC-3′ (*PDF1.2_F*) and 5′-AATCCATGGAATACACACGATTTAGCACC-3′ (*PDF1.2_R*); 5′-TTCCACAACCAGGCACGAGGAG-3′ (*PR1_F*) and 5′-CCAGACAAGTCACCGCTACCC-3′ (*PR1_R*). The AGI codes were as follows: *CHIB* (AT3G12500), *PDF1.2* (AT5G44420), *PR1* (AT2G14610), and *AtACT2* (AT3G18780). The control for equal loading was *AtActin* using the primers 5′-GTTAGCAACTGGGATGATATGG-3′ and 5′-CAGCACCAATCGTGATGACTTGCCC-3′. Candidate priming genes were PCR-amplified from 100 ng of cDNA using an annealing temperature of 60°C. Amplified PCR products were separated by 2% agarose gel electrophoresis. The qRT-PCR assays were performed using a Chromo4 Real-Time PCR system (Bio-Rad). Reaction mixtures (10 μl) contained 5 μl of 2 × Brilliant SYBR Green qPCR master mix (Bio-Rad), cDNA, and 10 pM of each primer. Thermocycle parameters were as follows: initial polymerase activation for 10 min at 95°C, and then 40 cycles of 30 s at 95°C, 30 s at 60°C, and 42 s at 72°C. Conditions were determined by comparing the threshold values in a dilution series of the RT product, followed by a non-RT template control and a non-template control for each primer pair. Relative RNA levels were calibrated and normalized to the level of *AtAct2* mRNA.

### Statistical Analysis

Data were subjected to analysis of variance (ANOVA) using JMP software (SAS Institute Inc., Cary, NC, USA). Significance of *B. subtilis* GB03 VOC treatment effects were determined by the magnitude of the *F* value at *P* = 0.05. When a significant *F* value was obtained for treatments, separation of means was accomplished using Fisher’s protected least significant difference (LSD) at *P* = 0.05. Experiments done in four replicate and each experiment repeated for three time. The results of repeated trials of each experiment were similar. Therefore, one representative trial of each experiment is reported in the section “Results.”

## Results

### Assessment of Bacterial VOC-Mediated Plant Protection Against *B. cinerea* Infection

**FIGURE 1:**
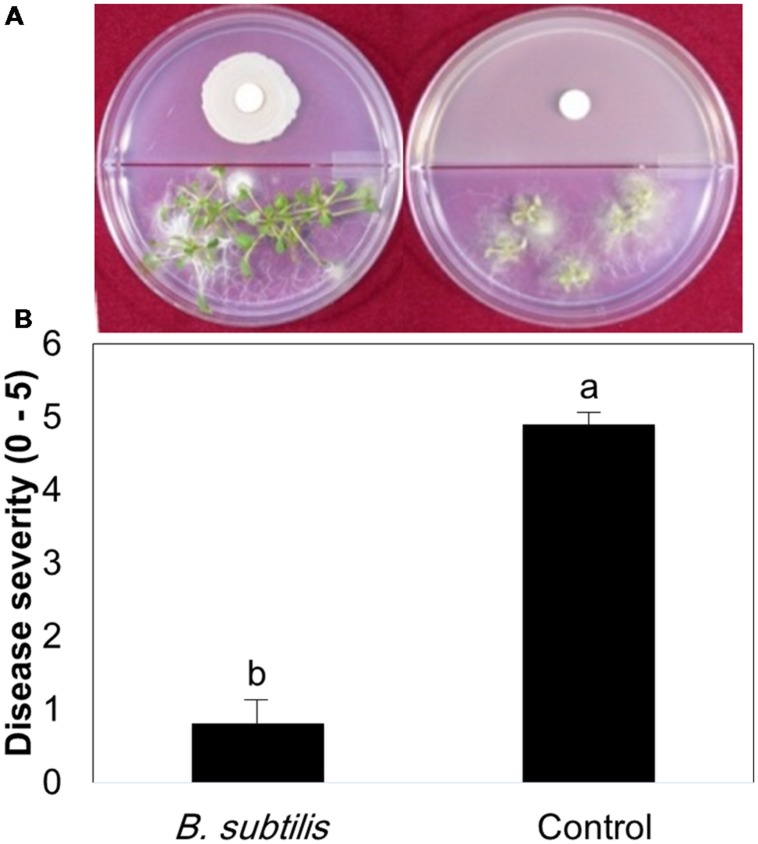
**Plant protection against *Botrytis cinerea* by volatile compounds emitted from *Bacillus subtilis* GB03.** Bacterial volatile compounds (BVCs) elicit induced systemic resistance (ISR) against *B. cinerea* infection. **(A)** Representative photo of ISR by BVCs using by the I-plate system. The right panel = BVC treatment; left panel = water control **(B)** Disease severity (0 = no symptom, 10 = severe necrosis) in the presence of bacterial VOCs and water-treated controls was recorded and plates were photographed 3 days after pathogen challenge. Different letters indicate significant differences between treatments according to Fisher’s LSD test at *P* = 0.05.

**FIGURE 2:**
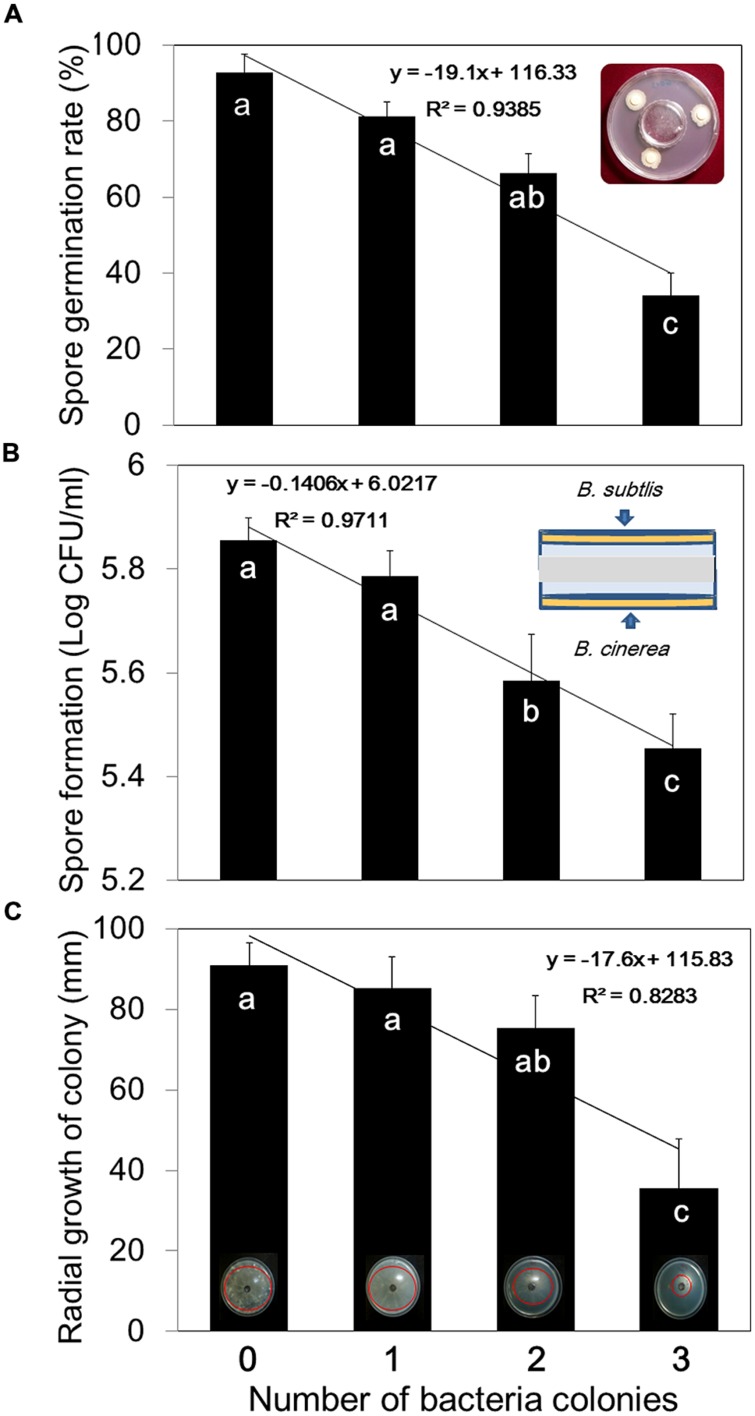
**Dose-dependent inhibition of *B. cinerea* mycelial growth, spore germination, and spore development by *B. subtilis* GB03 volatile compounds. (A)** Spore germination, **(B)** spore formation, and **(C)** mycelial growth of *B. cinerea* were measured after 7 days in the presence of one, two, or three spot-inoculated filter disks (5 mm diameter) of *B. subtilis* GB03 bacterial suspension (10^8^ CFU/ml) that were allowed to grow for 24 h before addition of fungi. Different letters indicate significant differences between treatments according to Fisher’s LSD test at *P* = 0.05. Error bars indicate standard error of the mean (SEM).

We tested the effects of bacterial VOCs on different stages of *B. cinerea* growth, including spore germination, mycelial growth, and spore production. First, we confirmed that pretreatment of plants with bacterial VOCs conferred protection against subsequent *B. cinerea* infection (**Figure 1**). A few leaves pretreated with *B. subtilis* GB03 VOCs developed very mild scars at the fungal inoculation site, whereas control leaves developed severe necrosis (**Figure 1**). Bacterial VOCs suppressed fungal growth and development at all phenological stages in a dose-dependent manner (**Figures 2A–C**). In all of these tests, inoculation of one filter paper disk with bacteria, which represented a low dose of VOCs, did not significantly affect mycelial growth, spore germination, and spore production (**Figures 2A–C**). Exposing *B. cinerea* to VOCs from two inoculated disks did not significantly affect mycelial growth and spore germination (**Figures 2A,C**), but reduced spore production by 0.271 log CFU/ml (**Figure 2B**). Exposing fungi to VOCs from three inoculated disks reduced mycelial growth, spore germination, and spore production by 61, 64, and 7%, respectively (**Figure 2**). When the bacteria colonized the entire Petri dish, fungal growth was completely inhibited (data not shown).

### Differentiation of Direct versus Indirect Mechanisms of Plant Protection against Pathogen Infection by Bacterial VOCs

**FIGURE 3:**
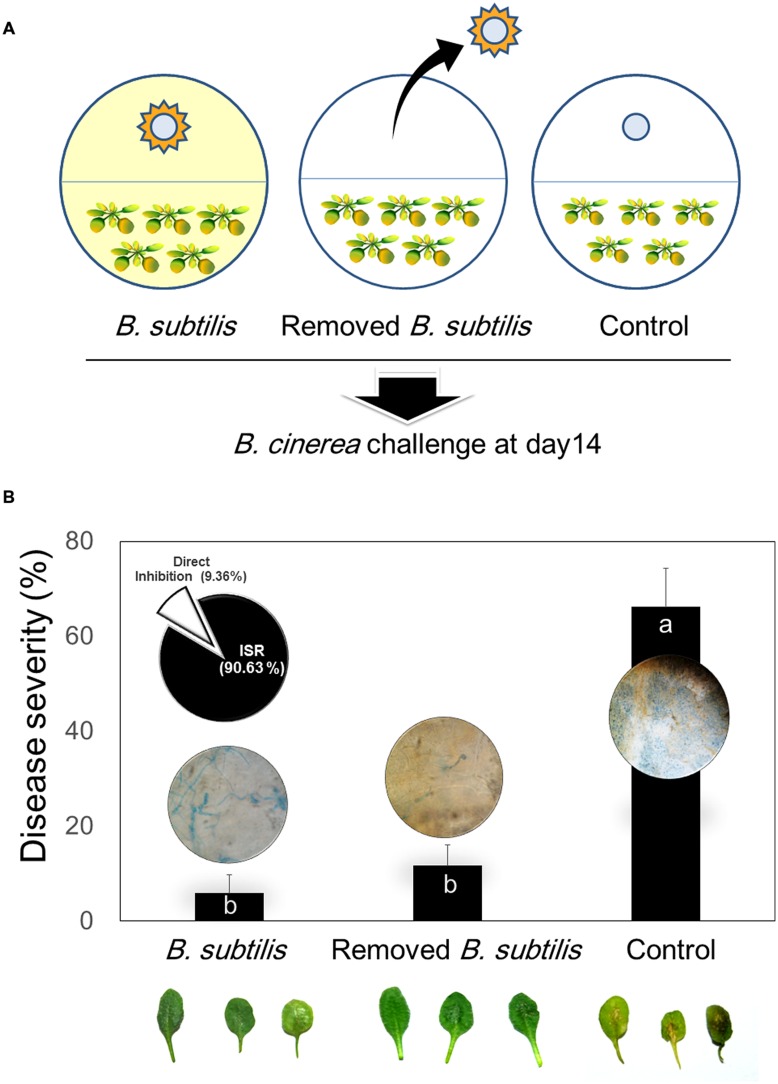
**Direct and indirect mechanisms of *B. subtilis* GB03 volatile compound effects on *Botrytis cinerea* and ISR of *Arabidopsis*. (A)** Schematic of experimental design to test bacterial VOC-elicited ISR using the two-chamber I-plate system. *Arabidopsis* seedlings were grown in one chamber and *B. subtilis* GB03 were grown in the other chamber. The two chambers share the same head space, which allows bacterial volatiles to be transmitted to seedlings and inoculated fungal pathogens. In one group of plates, *B. subtilis* GB03 were removed before fungal inoculation at 14 days (middle image); in the other group, bacteria and volatile emission were retained (left image). Water-treated filter disks were used as control. **(B)** Disease severity caused by *B. cinerea* inoculation on *Arabidopsis* seedlings pretreated with *B. subtilis* GB03 VOCs, and in the presence and absence of continuing *B. subtilis* VOC emission. The inset pie graph indicates percentage plant protection conferred by VOC-elicited ISR (indirect mechanism) and by VOC-induced inhibition of fungal growth (direct mechanism). The inset photos above or inside bar graph indicate that *B. subtilis* GB03 volatile compounds inhibit leaf attachment of *B. cinerea.* Fungi growth were checked after staining with Trypan blue. Different letters indicate significant differences between treatments according to Fisher’s LSD test at *P* = 0.05.

We designed experiments to differentiate between direct inhibition of fungal growth by VOCs and indirect inhibition by VOC induction of plant systemic resistance. For these experiments, we used one filter disk inoculated with *B. subtilis* GB03. *Arabidopsis* seedlings were transplanted into one chamber of an I-plate and the other chamber contained TSA medium with one disk inoculated with *B. subtilis* GB03 or control disk with 30 μl of distilled water. Plates were incubated in a growth chamber for 14 days. The plates containing GB03 were separated into two groups. In the first group, the TSA medium and disk containing bacteria were removed immediately before pathogen challenge. These plates no longer contained bacterial VOCs, and were designated as the “*B. subtilis*-removed” group (**Figure 3A**). In this group, only plant ISR could function against pathogen infection because there were no longer any VOCs to confer direct plant protection against *B. cinerea* colonization. In the second group, the bacteria were not removed (designated as “*B. subtilis*-treated”); therefore, there was continuous production of volatiles, which could induce indirect fungal inhibition by eliciting plant ISR and direct antagonism (**Figure 3A**). Based on M&M formula, ISR proportion was 90.63% and direct inhibition of fungi was 9.36% which mean that ISR have main role in suppression of *B. cinerea* on *Arabidopsis* in low concentration of VOCs (**Figure 3B**). Monitoring *in vivo* growth of *B. cinerea* on *Arabidopsis* by quantitative measurement of β*-tubulin* expression revealed that fungal growth inhibition was primarily due to stimulation of plant defense responses. There were differences between β*-tubulin* expressions in the “*B. subtilis*-removed” and “*B. subtilis*-treated” groups within 10 h post-infection (hpi). However, these effects were temporally unstable and were not significantly different at 20 and 30 hpi. There were no differences in β*-tubulin* expression levels between the “*B. subtilis*-removed” and “*B. subtilis*-treated” groups in 20 and 30 hpi.

### Effects of Bacterial VOCs on artificial Surface and Leaf Attachment of *B. cinerea*

**FIGURE 4:**
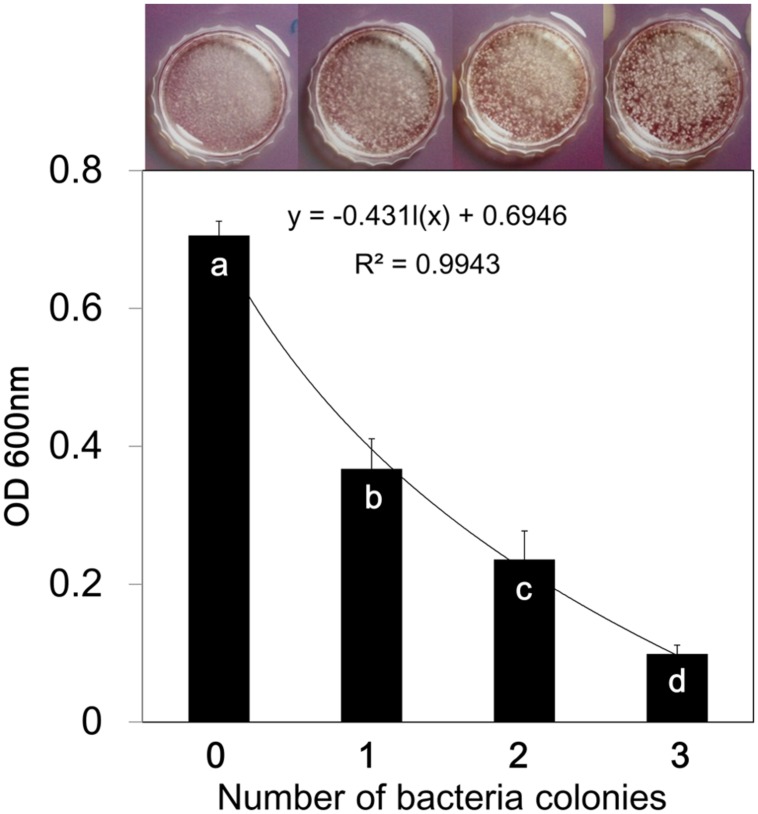
***Bacillus subtilis* GB03 volatile compounds inhibit surface biofilm formation of *Botrytis cinerea*.** Fungal biofilm formation in the presence of different concentrations of bacterial VOCs was measured using the CV staining method. The absorbance of CV retained in the fungal biofilm was measured spectrophotometrically at 600 nm, and represents the biofilm quantity. Fungal cultures were photographed 7 days after the start of VOC exposure. Different letters indicate significant differences between treatments according to Fisher’s LSD test at *P* = 0.05.

We performed microscopic investigation of *B. cinerea* colonization of *Arabidopsis* leaves. The results revealed that the “*B. subtilis*-treated” group displayed more aggressive epiphytic growth on leaf surfaces than that of the “*B. subtilis*-removed” group (**Figure 3B**). These experiments were performed using one filter disk inoculated with *B. subtilis* GB03; however, one inoculated disk did not significantly affect spore germination and mycelial growth. We concluded that VOCs might interfere in mycelial attachment to the leaf surface, and this could cause epiphytic mycelial growth and inability to penetrate and colonize host tissue. We investigated mycelial surface attachment in the presence of bacterial VOCs using a universal biofilm formation method (Mowat et al., 2007). The results showed that VOCs emitted from one disk inoculated with GB03 were sufficient to reduce mycelial attachment to the hydrophobic Petri dish surface by 51%. In the presence of VOCs emitted from three inoculated disks, mycelial surface attachment was reduced by sevenfold compared with that of the control (**Figure 4**). However, Higher VOC levels increased the air-liquid interface biofilm which means that fungi colonized surface of liquid media (**Figure 4**). A possible explanation for this aerotactic response could be an increase in mycelial hydrophilicity. Talbot et al. (1996) reported that production of cutinase and SC3 hydrophobin increased mycelial hydrophilicity. Therefore, it was possible that VOCs reduced mycelial attachment to the hydrophobic cuticular surface of the leaf. Further work is needed to confirm VOC effects on surface attachment regulation in *B. cinerea*. Our observations support the hypothesis that VOCs increase epiphytic growth of *B. cinerea* on *Arabidopsis* leaves. Although low VOC concentrations do not significantly affect fungal growth, they can affect some virulence factors such as host penetration (**Figure 3**).

### Bacterial VOC-Mediated Priming of Plant Defense Against *B. cinerea* Infection

**FIGURE 5:**
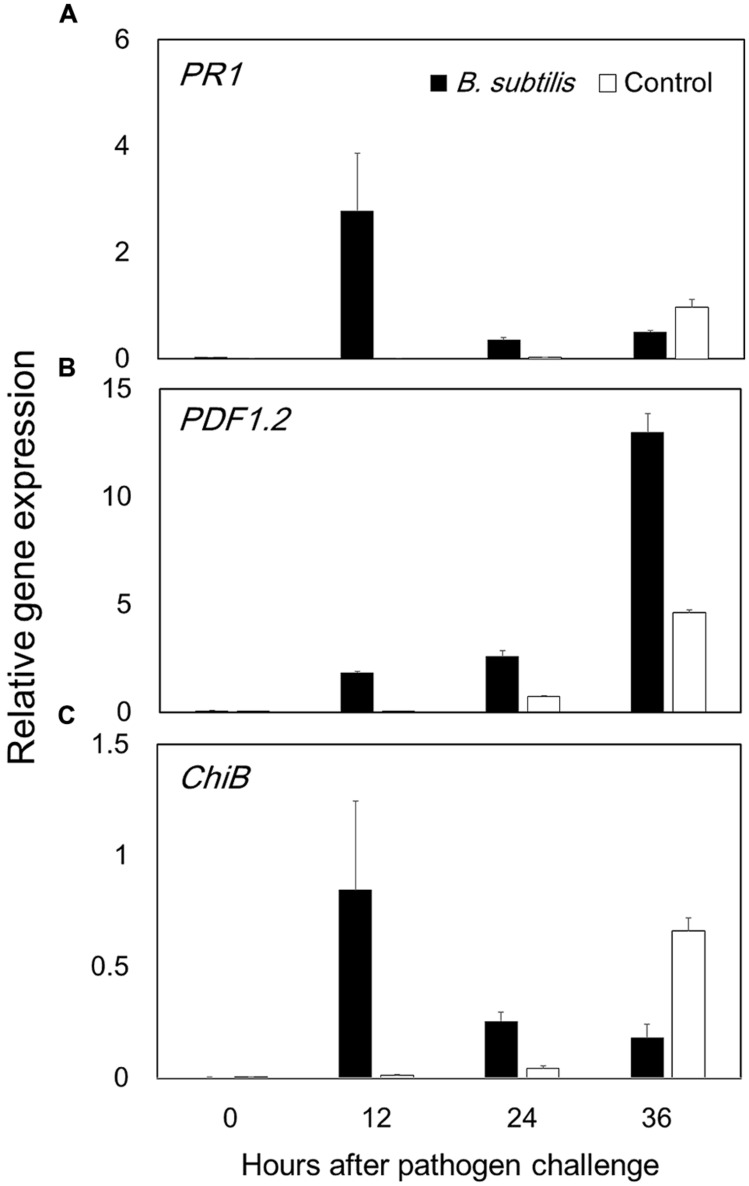
***Bacillus subtilis* GB03 volatile compounds elicit *Arabidopsis* defense priming of the jasmonic acid and salicylic acid signaling pathways.** Defense priming gene expression levels were measured by time-course qRT-PCR analysis of **(A)** *PR1* in the salicylic acid signaling pathway, **(B)** *PDF1.2* in the jasmonic acid signaling pathway, and **(C)** *ChiB* in the ethylene signaling pathway **(C)**. The ratio of gene expression in the *B. subtilis* GB03-treated plants versus that in the water-treated control relative to expression of the *Actin* gene is computed as the mean ± SEM. Different letters indicate significant differences between treatments **(A,B)** according to Fisher’s LSD test at *P* = 0.05.

Volatile organic compounds-mediated plant defense priming was investigated using qRT-PCR analysis. The results indicate that JA signaling has a key role in VOC-elicited plant defense responses, because *PDF1.2* expression was much higher in VOC-treated plants than in control plants. Maximum increases in *PDF1.2* expression levels in VOC-treated and control plants were 13 and 4.6-fold, respectively, at 36 hpi (**Figure 5B**). *PR-1* expression, which is a marker of SA signaling, had a maximum 2.8-fold increase in VOC-treated plants at 12 hpi (**Figure 5A**). The activation of *PDF1.2* and *PR-1* at 36 and 12 hpi, respectively, represents early stages of disease establishment and plant defense priming. *CHIB* expression, which is a marker ET signaling, did not display statistically significant differences in VOC-treated and control plants (**Figure 5C**). This result is not in agreement with our previous results (Ryu et al., 2004), and other ET-dependent marker genes should be analyzed. Previous studies report that a necrotrophic pathogen activated the JA signaling pathway and suppressed SA-dependent pathways (Pieterse and Van Loon, 2007), and activation of one pathway (JA or SA) had antagonistic effects on the other (Pieterse et al., 2009). However, the present study indicates that GB03 VOCs activate both pathways.

## Discussion

The debate on direct and indirect effects of bacterial VOC-mediated plant protection against pathogens arises because bacterial volatile compounds can directly inhibit pathogen growth (Chaurasia et al., 2005; Vespermann et al., 2007; Chen et al., 2008; Yuan et al., 2012) and indirectly elicit plant immune responses against target pathogens (Lee et al., 2012; Song and Ryu, 2013; Choi et al., 2014). Studies on direct effects of volatile compounds generally utilize very high volatile concentrations, which do not occur under natural conditions. Pedigo (1999) mentioned that high concentrations of any chemical can induce toxic effects. Studies on indirect effects of volatile compounds utilized low VOC concentrations by exposing plants and pathogens to one filter paper disks (5 mm diameter) with confluent PGPR growth (Ryu et al., 2004; Lee et al., 2012). In present study, one filter disk did not significantly affect mycelial growth, spore germination, and spore production, but reduced disease severity by 60.26% (**Figures 1** and **2**). This indicates that VOC-elicited plant ISR is involved in plant protection when VOC concentrations are low. Park et al. (2015) shown that one colony of bacteria was enough to increase plant growth up to 80% in natural condition. They revealed that 5 ng of 2-butanone is enough for promote plant growth. However, three filter paper disks significantly reduced mycelial growth, spore production, and spore germination (**Figure 2**). Exposing fungi to whole Petri dishes with confluently growing *B. subtilis* GB03 abolished mycelial growth. These results indicate that high VOC concentrations could be toxic for fungi. By contrast, treatment with low VOC concentrations (Ryu et al., 2004) or low levels of individual volatiles such as 0.2 pg of 2,3 butanediol (Ryu et al., 2004), 10 μM of MeJA (Jiang et al., 2015; Wang et al., 2015), 1 ppm of 2,4-Di-*tert*-butylphenol (Sang and Kim, 2012), 0.001 ppm of acetoin (Ann et al., 2013) or 100 μM hexadecane (Park et al., 2013) reveal the indirect mechanism of VOC-elicited plant ISR. In our previous work, we showed that 100 μM of pure volatiles acetoin, 2,3 butanediol, 3-pentanol and 1-pentanol had no negative effect on growth of *B. cinerea* but suppressed disease on *Arabidopsis* (Sharifi et al., 2013).

The current study showed that VOC-elicited plant defense responses continued to be active after the bacteria and their emitted volatiles were removed, and plant immunity to infection was still observed at the time of pathogen challenge (**Figure 3B**). We suggests that direct VOC inhibition of fungal growth is only responsible for a small percentage (9.36%) of the total VOC effect in suppressing *B. cinerea*. Microscopic inspection showed that volatiles have effect on surface attachment of *B. cinerea*. In the presence of VOCs emitted from one inoculated filter disks, fungi growth more epiphytic (**Figure 3**). However, expression of β*-tubulin* showed that there were difference in colonization between *B. subtilis* and removed *B. subtilis* just in 10 h. There was no difference in leaf colonization in the presence or absence of VOCs at 20 and 30 hpi. Bacterial VOCs emitted from one filter disk were sufficient to reduce *B. cinerea* attachment to the leaf surface (**Figure 4**). This could suppress fungal penetration and induce epiphytic growth on the leaf surface (Talbot et al., 1996; Tucker and Talbot, 2001). Biotrophic and necrotrophic fungi need to tightly attach to the host cuticular surface and then penetrate the host tissue. Attachment to the host surface during the initial stage of fungal pathogenesis is crucial for fungal establishment on the host (Talbot et al., 1996; Tucker and Talbot, 2001; Harding et al., 2009). Altering the surface attachment efficiency of *B. cinerea* reduced or inhibited its pathogenicity (Doss et al., 1995; Epstein and Nicholson, 1997). Quintana-Rodriguez et al. (2015) shown that volatiles emitted from diseased plant could increase resistance in neighbor susceptible plants. These volatiles also were able to directly inhibited conidia germination *in vivo* and *in vitro* but in dose dependent manner.

We evaluated VOC-elicited plant defense priming by performing a time-course qRT-PCR analysis of defense marker gene expression (**Figure 5**). We analyzed the expression of *PDF1.2*, *PR-1*, and *ChiB*, which are involved in JA, SA, and ET signaling pathways, respectively. The highest gene expression achieved in *PDF1.2* after *B. subtilis* GB03 treatment.*PDF1.2* expression reached maximum at 36 hpi, which is within the typical plant defense priming temporal window. The JA signaling pathway is the primary pathway induced by necrotrophic fungi such as *B. cinerea* (Glazebrook, 2005). Our previous work revealed that Me-JA could suppress *B. cinerea* disease on *Arabidopsis* up to 40% (Sharifi et al., 2013). *PR-1* expression also displayed evidence of defense priming, with maximum expression occurring at 12 hpi. The present study observed that *B. subtilis* GB03 VOCs activated both JA and SA signaling pathways. Several studies report that *Bacillus* strains can simultaneously activate JA and SA pathways, and SA, JA, and ET pathways (Buensanteai et al., 2009; Rudrappa et al., 2010; Niu et al., 2011; Lee et al., 2012; Choi et al., 2014). The *B. subtilis* volatile compound acetoin activated expression of both *PR-1* and *PDF1.2* (Rudrappa et al., 2010). Tridecane from *Paenibacillus polymyxa* E681 induced *PR-1* and *VSP2* expression (Lee et al., 2012).

## Conclusion

We intended to link information from two types of reports. Researcher who mentioned that, volatiles are responsible for direct fungi growth inhibition and researchers who report that, ISR is mechanism of volatiles action. The optimum VOCs concentration that did not inhibit the fungus *in vitro* still elicit plant defense strategies to prevent subsequent *Botrytis* infection (**Figures 2** and **4**). Low VOCs concentrations elicit plant ISR, which is an indirect mechanism that results in 90.63% of disease suppression (**Figure 3**). By contrast, direct inhibition of fungal growth and development by low VOC concentrations accounts for 9.36% of the total disease suppression (**Figure 3**). The direct mechanism may become more prominent with higher VOC concentrations in a dose-dependent manner. JA was the main signaling pathway in activation of plant defense by means of *B. subtilis* GB03 volatiles (**Figure 5**). However, there were no cross-talk between SA and JA signaling pathway as expression of both genes increased.

## Author Contributions

RS conceived and performed experiments, interpreted data and contributed to the drafting of the manuscript. CMR gave experimental advice, interpreted data, and contributed to the drafting of the manuscript. All authors contributed to the discussion and approved the final manuscript.

## Conflict of Interest Statement

The authors declare that the research was conducted in the absence of any commercial or financial relationships that could be construed as a potential conflict of interest.
